# Human NCU-G1 can function as a transcription factor and as a nuclear receptor co-activator

**DOI:** 10.1186/1471-2199-8-106

**Published:** 2007-11-16

**Authors:** Knut R Steffensen, Mariam Bouzga, Frode Skjeldal, Cecilie Kasi, Almira Karahasan, Vilborg Matre, Oddmund Bakke, Sylvain Guérin, Winnie Eskild

**Affiliations:** 1 Department of Molecular Biosciences, University of Oslo, Norway; 2 Department of Biosciences and Nutrition, Karolinska Institutet, Huddinge, Sweden; 3 Oncology and Molecular Endocrinology Research Center, CHUL, Centre Hospitalier Universitaire de Québec and Laval University, Québec, Canada

## Abstract

**Background:**

Novel, uncharacterised proteins represent a challenge in biochemistry and molecular biology. In this report we present an initial functional characterization of human kidney predominant protein, NCU-G1.

**Results:**

NCU-G1 was found to be a highly conserved nuclear protein rich in proline with a molecular weight of approximately 44 kDa. It is localized on chromosome 1 and consists of 6 exons. Analysis of the amino acid sequence revealed no known transcription activation domains or DNA binding regions, however, four nuclear receptor boxes (LXXLL), and four SH3-interaction motives in addition to numerous potential phosphorylation sites were found. Two nuclear export signals were identified, but no nuclear localization signal. In man, NCU-G1 was found to be widely expressed at the mRNA level with especially high levels detected in prostate, liver and kidney. Electrophoretic mobility shift analysis showed specific binding of NCU-G1 to an oligonucleotide representing the footprint 1 element of the human cellular retinol-binding protein 1 gene promoter. NCU-G1 was found to activate transcription from this promoter and required presence of the footprint 1 element. In transiently transfected Drosophila Schneider S2 cells, we demonstrated that NCU-G1 functions as a co-activator for ligand-activated PPAR-alpha, resulting in an increased expression of a CAT reporter gene under control of the peroxisome proliferator-activated receptor-alpha responsive acyl-CoA oxidase promoter.

**Conclusion:**

We propose that NCU-G1 is a dual-function protein capable of functioning as a transcription factor as well as a nuclear receptor co-activator.

## Background

Vitamin A is required for proper cell growth, differentiation and function. These processes depend on expression of appropriate genes at the right time and place, and in correct amounts. The biological activity with regard to vitamin A control of gene expression is carried out by retinoic acid (all-trans isomer or 9-cis isomer), the ligand for retinoic acid receptors (RARs and RXRs), which are members of the nuclear receptor superfamily of ligand-activated transcription factors [1,2]. Malfunction of vitamin A regulated genes have been described in various cancer types [3-5]. Some human carcinomas were shown to be affected in this manner due to reduced levels of RAR-β_2_, a retinoic acid receptor isoform involved in negative growth regulation [6].

In addition to suboptimal expression or malfunctioning of retinoic acid receptors, reduced retinoic acid (RA) activity may also occur because RA is not available. Expression of some retinoic acid receptors is itself vitamin A dependent, hence the availability of RA becomes crucial. Most cells depend on conversion of retinol to RA to satisfy their needs for this ligand [7]. Retinol (ROH) is taken up from circulating retinol-binding protein (RBP) or released from intracellular storage of retinylester and transferred to cellular retinol-binding protein type 1 (CRBP1) which regulates its metabolism. In addition to regulating cellular uptake of ROH, CRBP1 presents ROH to lecithin:retinol acyl transferase (LRAT) for esterification and storage in lipid droplets in the cell cytoplasm or interacts with oxidizing enzymes which convert ROH to RA. Hence, CRBP1 plays an essential role in the regulation of vitamin A controlled genes and maintenance of proper cell health.

In contrast to previous concepts of CRBP1 as an inert chaperone for ROH, CRBP1 is now viewed as an active participant in vitamin A metabolism [8-10]. The general understanding of the mechanisms regulating CRBP1 expression and function, however, is not very comprehensive. It has been reported that retinoids, lipids and serum factors increase the expression of CRBP1, whereas cAMP and glucocorticoids reduce expression [11-16]. Presently it is not known whether any regulation of CRBP1 activity by way of posttranslational modification is taking place. We are working to identify regulatory mechanisms controlling CRBP1 expression by identifying novel proteins interacting with the proximal 5'-region (-567/+104) of the human CRBP1 gene. In a previous report we identified the transcription start site and seven DNA elements (FP1 – FP7) which specifically bind nuclear proteins from liver, kidney and prostate [17]. Several of the DNA elements were potential binding sites for novel transcription factors. Here we report the identification and characterisation of one such protein, NCU-G1, which interacts specifically with FP1 and stimulates transcription from the CRBP1 promoter. In addition, NCU-G1 functions as a nuclear receptor co-activator by stimulating the transcriptional activity of peroxisome proliferator-activated receptor-alpha (PPAR-alpha).

## Results

### Cloning of human NCU-G1

Previous studies of the hCRBP1 gene promoter identified a DNA-element (FP1, +66/+96) that comprises target sites for both the nuclear factor 1 (NF1) and specificity protein 1 (Sp1) transcription factors [17]. Further studies, using SDS-PAGE fractionation and partial renaturation of nuclear proteins, enabled us to detect binding of two unknown proteins, designated Bp1 and Bp2, to the FP1-element [18]. Bp1 and Bp2, which compete with both Sp1 and NF1 for binding to the Fp1 element, could be revealed by exploiting the fact that neither Sp1 nor NF1 renature after SDS-PAGE fractionation and hence do not bind FP1 in electrophoretic mobility shift assay (EMSA) [19].

**Figure 1:**
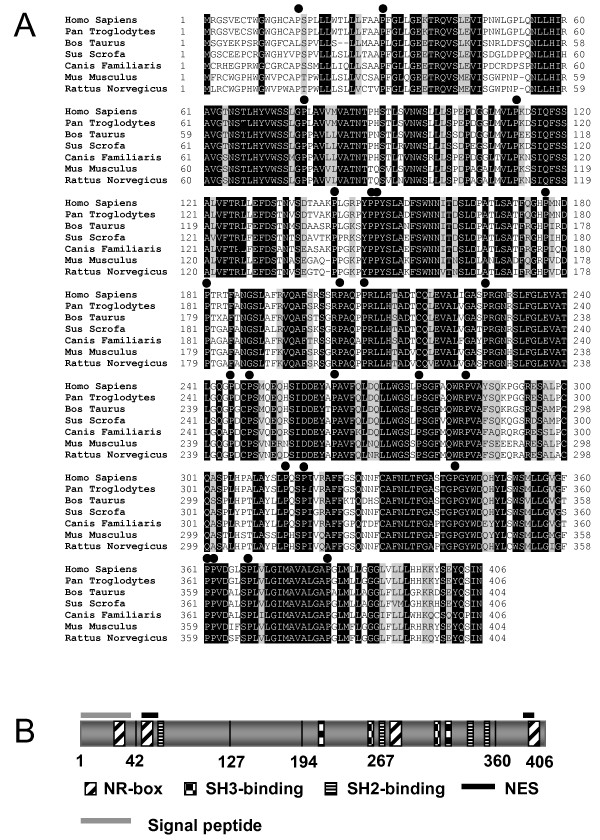
**Analysis of the NCU-G1 amino acid sequence**. Panel A: Comparison of deduced NCU-G1 amino acid sequences from various species. Totally conserved amino acids are shown against a black background. Highly conserved amino acids are shown against a grey background. The positions of conserved prolines are marked with black bullets. Panel B: Potential functional motifs in human NCU-G1.

In order to study these proteins in more detail, we used the One-Hybrid cloning strategy to clone their corresponding cDNAs from an expression library prepared from human placenta. The FP1 element was used as bait after some modifications to avoid recognition by NF1 and Sp1. Screening of the placenta expression library resulted in isolation of three unique clones one of which contained an insert of 1.7 kb. DNA sequencing of this clone revealed an open reading frame of 1221 base pairs yielding a protein of 406 amino acids (figure 1A). Blast analysis identified it as kidney predominant protein, alias NCU-G1 or C1orf85 homologous to the previously published mouse NCU-G1 [20]. A search of the human genome database showed that NCU-G1 is located on chromosome 1, 1q23.1, locus 112770, and has an open reading frame spanning 6 exons. No human homologues were identified, indicating that NCU-G1 is unique.

Comparison with NCBI GenBank [21] showed a number of previously submitted sequences corresponding to human NCU-G1 (GenBank: NM_144580, BC018757, BC011575), however, no functional characterization of this protein has been reported. Database searches including other species revealed that mammals, birds, frogs and certain mosquitos all have an NCU-G1 gene (figure 1A), whereas *Drosophila melanogaster *does not. In rodents (mouse, rat) the open reading frame (ORF) is 404 amino acids. The human gene has inserted a leucine after amino acid 52 and a lysine after amino acid 139. The NCU-G1 gene appears to be highly conserved. Complete conservation of more than 25% of amino acids is seen from mosquito to man, and conservative conservation is found in all available sequences from worm to man. At the amino acid level, homologies between man/chimpanzee is 99.8%, man/rat 79%, man/mouse 79% and rat/mouse 82%. Figure 1A shows that there are highly conserved regions and distinct areas where variations can occur. The protein has a high content of proline (9.1%) but no specific proline-rich regions. However, the proline positions are well conserved.

**Figure 2:**
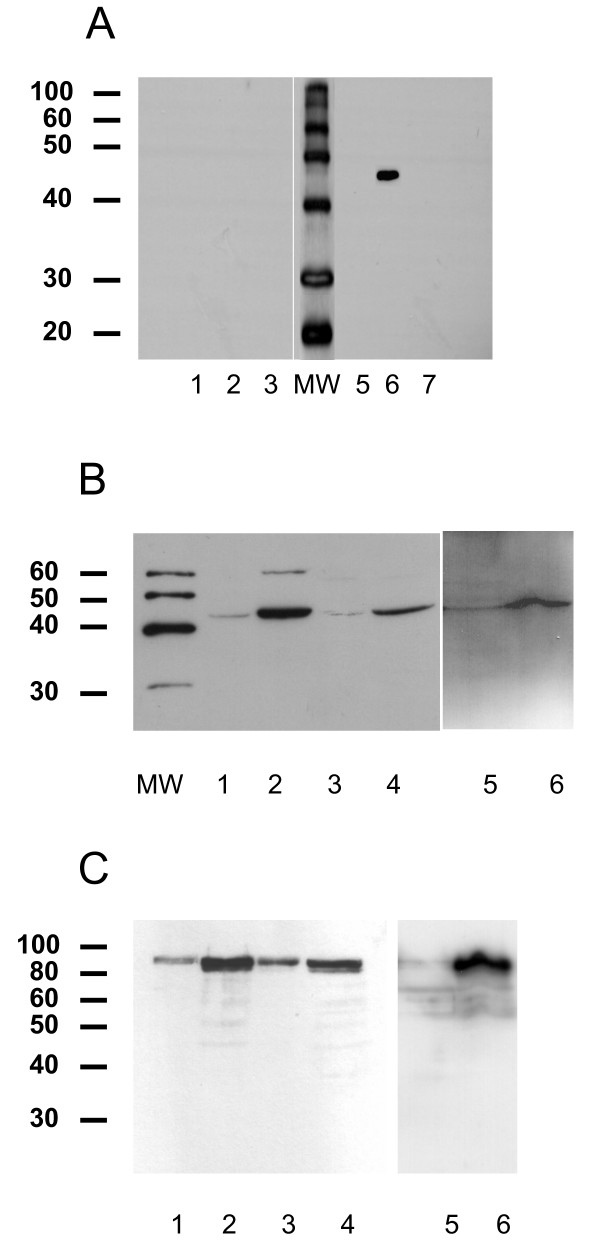
**Endogenous NCU-G1 is a nuclear protein**. Panel A: S2 cells were transfected with expression vectors for hNCU-G1 (pAc-NCU-G1) or its truncated version pAc-hNCU-G1(Δ exon 6). Whole cell extracts (20 μg/lane) were analyzed by Western blotting using preimmune serum (lanes 1 to 3) or an antiserum raised against a C-terminal 15 amino acid peptide (lanes 5 to 7). Lane 1 and 5: control, untreated S2 cells, lane 2 and 6: S2 cells expressing hNCU-G1, lane 3 and 7: S2 cells expressing hNCU-G1(Δ exon6). Panel B: Cytoplasmic and nuclear extracts (20 μg/lane) from JEG3, RPE and 293 cells were size-fractionated on 10% SDS-PAGE gels and analyzed by Western blotting using NCU-G1 antiserum. Lane 1: JEG3 cytosol, lane 2: JEG3 nuclear extract, lane 3: RPE cytosol, lane 4: RPE nuclear extract, lane 5: 293 cytosol, lane 6: 293 nuclear extract. Panel C: The membranes used in panel B were stripped and analyzed with an antibody against Sp1.

To search for information on the structure and function of the NCU-G1 gene product, a variety of data mining tools were used. NCU-G1 has a predicted MW of 43.8 kDa and an acidic pI of 6.1 [22]. The molecular weight was verified by Western analysis using a polyclonal antibody which we raised against a 15-amino acid peptide covering the C-terminal sequence (see below, figure 2A). No homologies to known DNA-binding motifs, transcription activation domains or regions with enzymatic activity were found. Somewhat surprising, NCU-G1 has four NR-boxes with the LXXLL signature sequence [23,24]. Such NR-boxes are found in a number of transcriptional co-activators, such as the p160-family of co-activators for which the LXXLL sequences are characteristic [25,26]. The presence of an NR-box has been determined as necessary and sufficient for interaction with nuclear receptors, although both recruitment and effect on transcription are influenced by other factors such as the sequences flanking the NR-box, the ligand types of the nuclear receptor itself and the DNA response elements present on the target genes. As shown in figure 1B, the LXXLL-motifs were distributed over the entire protein. The first NR-box (aa 21–25) is conserved in all NCU-G1 sequences from man to frog. The second NR-box (aa 53–57) is unusual being preceded by a proline residue. This NR-box is found only in the NCU-G1 protein from man and chimpanzee. One of the inserted amino acids (aa 53), making up part of the difference between rodents and man, is the first leucine in NR-box no. 2 of man. The third NR-box (aa 267–271) is present in all mammals while the fourth NR-box (aa 390–394) is found in all mammals except in pig.

Four potential Src-homology 3 (SH3) interacting domains with the signature sequence PXXP were also identified in NCU-G1 (figure 1B). According to Cesareni *et al. *[27] motif no. 1 (aa 203–206) is a class II motif, whereas motif no. 4 (aa 298–301) belongs to class I. As shown for the modulator of non-genomic action of estrogen receptor (MNAR), these motifs may bind SH3 domains in c-Src, stabilizing its interaction with a nuclear receptor and leading to further activation of the ligand-bound receptor [28]. It may also activate the MAP kinase signal transduction pathway.

NCU-G1 was initially identified in nuclear fractions and therefore might be expected to have a nuclear localization signal (NLS), however, analysis of the NCU-G1 sequence failed to identify any candidate sequence for an NLS [18]. In spite of this, and as figure 1B indicates, two potential nuclear export signals (NES) conforming to the L(X_1–3_)L(X_2–3_)LXL formula characteristic of CRM1-mediated transport were found [29]. NES1 is localized in the N-terminus comprising amino acids 50 to 59 while NES2 is localized in the C-terminus comprising amino acids 383 to 392. Interestingly, both NES1 and NES2 overlap with an NR-box, no. 2 and no. 4, respectively.

### Human NCU-G1 is a nuclear protein

Our previous studies have shown that NCU-G1 is present in nuclear extracts but we could not exclude the possibility that it may also be present in other subcellular compartments [18]. The small size of NCU-G1 would predict an ability to shuttle between cytosol and nucleus. This hypothesis was examined by analyzing nuclear and cytosolic fractions from several cell types by Western blotting.

A polyclonal antibody was raised against a peptide representing the last fifteen amino acids in the C-terminus of NCU-G1. Its specificity was verified by Western analysis of transiently expressed NCU-G1 in Drosophila Schneider S2 cells which do not express this protein. The predicted size of NCU-G1 is 43.8 kDa. As shown in figure 2 (panel A, lane 6) a protein of approximately 45 kDa in size is detected with this antibody. In contrast, a transiently expressed truncated version of NCU-G1 lacking exon 6 comprising the C-terminal end of the protein could not be detected (figure 2A, lane 7). Using this antibody, we found that NCU-G1 is primarily localized in the nuclei of JEG3, RPE and 293 cells (figure 2B; lanes 2, 4 and 6, respectively). The traces of NCU-G1 detected in the cytosol were probably due to leakage from the nuclei during preparation. In order to test this assumption, the membranes were stripped and analyzed with an antibody against Sp1 as a marker of nuclear proteins. Figure 2C shows that some Sp1 is found in the cytosolic fractions, supporting the notion that partial nuclear rupture during preparation may account for the NCU-G1 detected in cytosol.

### Human NCU-G1 binds specifically to the FP1-oligonucleotide

**Figure 3:**
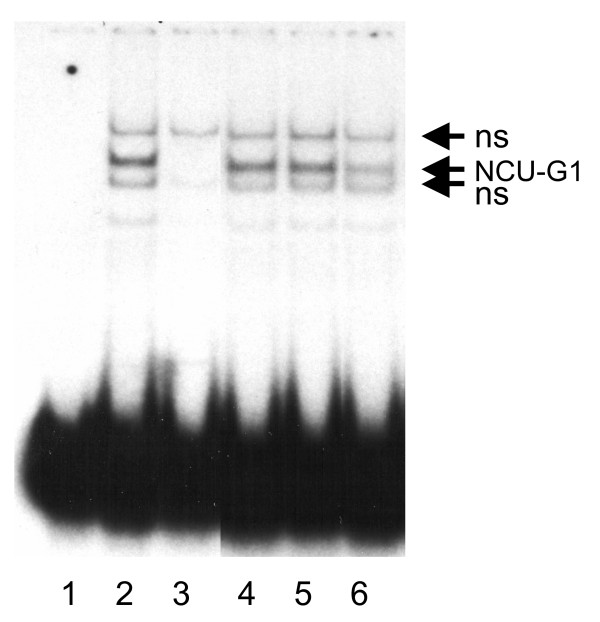
**Human NCU-G1 binds to the FP1-element of the CRBP1 promoter**. NCU-G1 was expressed as a fusion protein with GST in E. coli and purified. Interaction between GST-NCU-G1 and radiolabeled FP1-oligonucleotide was analysed by EMSA. Lane 1 shows ^32^P-labeled FP1 oligonucleotide (control). Lane 2 shows complex formation between GST-NCU-G1 and ^32^P-labeled FP1 oligonucleotide. Lane 3, 4, 5 and 6 show complex formation between GST-NCU-G1 and ^32^P-labeled FP1 oligonucleotide in the presence of 50-fold molar excess of unlabeled FP1 oligonucleotide, Ap1 oligonucleotide, Sp1 oligonucleotide or NF1 oligonucleotide, respectively. Ns = non-specific.

The initial identification of NCU-G1 was based on its ability to bind FP1 in EMSA. In order to show that our cloned NCU-G1 represents the proteins we set out to clone, its ability to bind DNA was studied. Figure 3 shows complex formation in EMSA between ^32^P-labeled FP1-oligonucleotide and GST-NCU-G1 fusion protein recombinantly expressed in E. coli. A major band (lane 2) shows specific binding which is inhibited in the presence of 50-fold molar excess of unlabeled FP1 oligonucleotide (lane 3). Unlabeled AP1-, Sp1- or NF1-oligonucleotides at 50-fold molar excess (lane 4, 5 and 6, respectively) are unable to inhibit complex formation. Control GST did not bind the FP1 oligonucleotide (data not shown).

### Human NCU-G1 stimulates transcription from the hCRBP1 promoter

**Figure 4:**
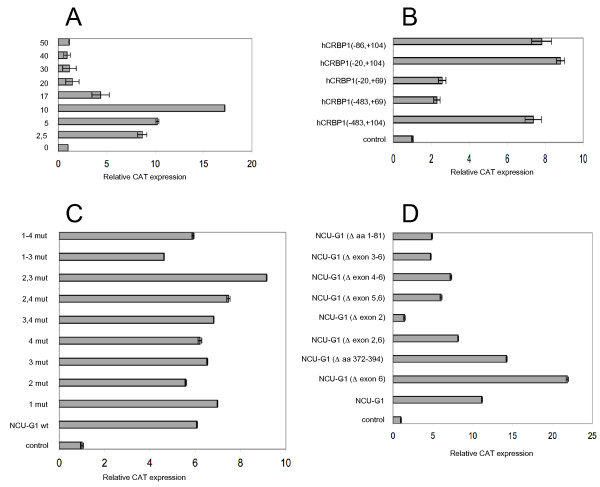
**Human NCU-G1 activates transcription from the hCRBP1 promoter**. Panel A: S2 cells were co-transfected with 250 ng pAc5.1/V5-His-LacZ, 350 ng phCRBP1-CAT and increasing amounts (2.5 ng – 50 ng) of pAc-hNCU-G1 as shown in the figure. Panel B: S2 cells were co-transfected with 250 ng pAc5.1/V5-His-LacZ, 350 ng plasmid expressing CAT under the control of various fragments of the hCRBP1 promoter as shown in the figure and 10 ng pAc-hNCU-G1. Panel C: S2 cells were co-transfected with 250 ng pAc5.1/V5-His-LacZ, 350 ng phCRBP1-CAT and 10 ng pAc-hNCU-G1 either wild-type or carrying mutated NR-boxes as shown in the figure. Panel D: S2 cells were cotransfected with 250 ng pAc5.1/V5-His-LacZ, 350 ng phCRBP1-CAT and 10 ng pAc-hNCU-G1 either wild-type or carrying various deletions as shown in the figure. Controls show expression of CAT in the absence of any NCU-G1. The cells were harvested 48 hrs after transfection. CAT was assayed by ELISA and β-galactosidase by enzyme activity. The data represent relative average values (pg CAT/unit β-galactosidase/hr +/- SE) from three independent experiments carried out in triplicates.

The ability of NCU-G1 to interact in EMSA with the FP1-element of the human CRBP1 promoter prompted us to analyse whether such interaction might entail an ability to modulate transcription. The human CRBP1 promoter (-567/+104 bp) was inserted upstream of the chloramphenicol acetyl-transferase (CAT) gene in a reporter vector (pCAT3) and transiently co-transfected with an expression vector for NCU-G1 into Drosophila Schneider S2 cells (S2 cells). These cells were chosen in order to avoid interference with Sp1 and NF1 which also bind to the FP1 element of the hCRBP1 promoter. Although these cells are not mammalian, S2 cells express transcription factors similar to those found in mammalian cells, but they often do not recognize mammalian proteins or mammalian DNA consensus elements, resulting in lower background and less interference [30]. Figure 4A shows that expression of low levels of hNCU-G1 leads to a 17-fold increase of CAT expression.

In order to test whether transcriptional induction was mediated by the FP1 element, we created deletion mutants of the CRBP1 promoter controlling reporter gene expression. The expression vector (pOCAT) used in these experiments has a somewhat higher basal activity resulting in the induction by NCU-G1 appearing smaller [17]. Expression of NCU-G1 increased reporter gene expression by 7.5-fold (fig. 4B). Deletion of FP1 from the full-length reporter construct (phCRBP1(-483/+69)OCAT) abrogated the induction of reporter gene expression by NCU-G1. Removing most of the promoter region upstream of the transcription start site but leaving FP1 intact (phCRBP1(-20/+104)OCAT) did not affect the ability of NCU-G1 to induce reporter gene expression. Again, a deletion that removes the FP1 element from this construct abolishes this induction (phCRBP1(-20/+69)OCAT). These results demonstrate that the FP1-element is an absolute requirement for NCU-G1's ability to induce reporter gene expression from the CRBP1 promoter.

In order to eliminate the possibility of any nuclear receptor (NR)-box contribution to the CRBP1 promoter controlled reporter gene expression, we created mutated versions of NCU-G1. Single or multiple NR-boxes were mutated from LXXLL to LXXAA to inhibit interaction with possible NR-box binding proteins. Figure 4C shows that mutation of one or more NR-boxes does not affect NCU-G1's ability to activate reporter gene transcription from the CRBP1 promoter, confirming that the NR-boxes are not involved.

Next, we studied the effect of deleting exons or segments of NCU-G1 on its ability to control reporter gene expression from the CRBP1 promoter. Figure 4D shows that deletion of exon 6 actually potentiates NCU-G1's stimulatory effect on CAT expression. This exon comprises a stretch of hydrophobic amino acids (aa 372–394) that may be involved in attenuating NCU-G1 stimulation of CAT expression. A deletion mutant lacking just this hydrophobic region, however, did not stimulate CAT expression to the same extent. Deletion of exon 5 and 6, exon 4–6, exon 3–6 or amino acid 1–81 reduces NCU-G1 stimulatory activity to approximately 50% of that of wild-type NCU-G1. In contrast, deletion of exon 2 results in a complete loss of stimulatory activity, indicating that this region comprising amino acids 42–126 is important for NCU-G1 activity.

### NCU-G1 mRNA is highly expressed in human liver, kidney and prostate

The tissue specific expression of NCU-G1 in man was investigated at the mRNA-level. Most tissues tested were found to express NCU-G1 mRNA, however, it was most abundant in liver, kidney and prostate, with somewhat lower levels in placenta, ovary and adrenal. In contrast, expression was low in heart, lung, and skeletal muscle (data not shown). These results are corroborated by data reported on the SAGE-express web-site [31].

### Human NCU-G1 acts as a co-activator for PPAR-alpha

The presence of four NR-boxes in NCU-G1 prompted us to study its possible capacity to function as a co-regulator for nuclear receptors, here represented by PPAR-alpha, in transient transfections of S2 cells. The rationale for choosing PPAR was the identification of a number of potential peroxisome proliferator-activated receptor response elements (PPARE) in the human CRBP1 promoter region, using "MatInspector" from Genomatix [23]. In addition, PPAR-beta agonists have been reported to stimulate CRBP1 expression [15]. The ability of NCU-G1 to stimulate PPAR transcriptional activity may therefore be relevant to CRBP1 expression.

**Figure 5:**
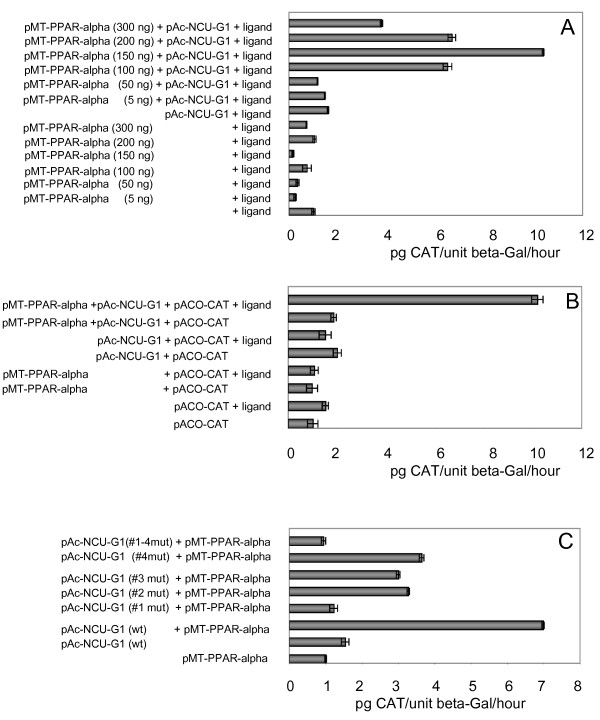
**Human NCU-G1 functions as a ligand dependent and NR-box dependent co-activator for PPAR-alpha in S2 cells**. Panel A: S2 cells were transiently transfected with 250 ng pAc5.1V5/His-LacZ, 350 ng of pACO-CAT, and increasing amounts of pMT-hPPARalpha, in the presence or absence of 10 ng of pAc-hNCU-G1. Twelve hrs after transfection WY14643 was added to 20 μM. The cells were harvested 56 hrs after transfection. CAT was assayed by ELISA and β-galactosidase by enzyme activity. The data represent relative average values (pg CAT/unit β-galactosidase/hr +/- SE) from three independent experiments carried out in triplicates. Panel B: S2 cells were transiently transfected with 250 ng pAc5.1V5/His-LacZ, 350 ng of pACO-CAT, together with pAc-hNCU-G1, pMT-hPPAR-alpha and WY14643 as indicated. The cells were treated and presented as in panel A. Panel C: S2 cells were transiently transfected with 250 ng pAc5.1V5/His-LacZ, 350 ng of pACO-CAT, 150 ng pMT-hPPAR-alpha as indicated and 10 ng of pAc-hNCU-G1, either wild-type or mutated as indicated in the figure. All cells were treated with 20 μM WY14643. The cells were harvested and data presented as in panel A.

We chose to use a reporter gene construct expressing CAT under control of the rat acyl-CoA oxidase gene promoter [32], a well-known PPAR-alpha target gene [33]. We first determined the optimal level of PPAR-alpha to be co-expressed with NCU-G1 in S2 cells. As shown in figure 5A, expression of CAT was very low when PPAR-alpha was expressed only in the presence of the PPAR-alpha agonist WY14643, nor did expression of NCU-G1 lead to any increase in reporter gene expression. When co-expressed, however, a strong induction of reporter gene expression was observed, indicating that NCU-G1 may function as a co-activator for PPAR-alpha. Maximal induction using 150 ng of the PPAR-alpha expressing vector increased reporter gene expression by 11-fold.

Interaction between co-activator NR-boxes and nuclear receptor AF-2 domains have been shown to require the ligand-induced conformational change of the latter [28]. Hence, we next sought to determine whether NCU-G1's ability to co-activate PPAR-alpha controlled reporter gene expression was ligand-dependent. Figure 5B shows that this is indeed the case. When both PPAR-alpha and NCU-G1 were expressed in the absence of the ligand, no CAT activity beyond the level directed by the pACO-CAT plasmid alone could be observed. However, addition of the agonistic ligand WY14643 increased CAT activity by approximately 11-fold. This result demonstrates that NCU-G1's ability to co-activate PPAR-alpha requires ligand activation of the nuclear receptor.

We next sought to determine which, if indeed any, of the four NR-boxes in NCU-G1 is responsible for the observed co-activation. As shown in figure 5C, mutation of NR-boxes 2, 3, or 4 lowered the co-activating capacity of NCU-G1 to approximately 50% of that observed with wild-type NCU-G1. Mutation of NR-box 1, however, completely abolished NCU-G1's ability to co-activate PPAR-alpha induced reporter gene expression, supporting our hypothesis that stimulation of PPAR-alpha controlled reporter gene expression by NCU-G1 is NR-box mediated. A similar effect was observed when all four NR-boxes were mutated. In summary, these results suggest that the mechanism of NCU-G1 co-activation of PPAR-alpha transcriptional activity requires an intact NR-box 1 and ligand mediated activation of the nuclear receptor.

### NCU-G1 co-localises with PPAR-alpha in the nucleus

**Figure 6:**
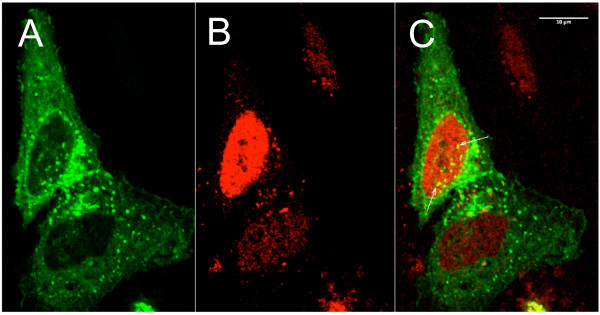
**Human NCU-G1 co-localises with PPAR-alpha in the nucleus**. HeLa cells were transiently transfected with 0.8 μg pNCU-G1-EGFP and 0.8 μg pRFP-PPAR-alpha. Twenty hours after transfection WY14643 was added to 2 μM. Six hours later the live cells were analysed by confocal fluorescence microscopy. Panel A: Green fluorescence showing subcellular localisation of NCU-G1-EGFP. Panel B: Red fluorescence showing subcellular localisation of RFP-PPAR-alpha. Panel C: Merged picture. Arrows show foci of co-localisation.

Nuclear receptor co-activators may be divided into a primary and a secondary group where members of the former interact directly with nuclear receptors and the latter interact with members of the primary group [34]. In an effort to determine which of these groups NCU-G1 may belong to, its ability to interact with PPAR-alpha was studied in mammalian as well as yeast two-hybrid systems. No interaction was detected indicating that NCU-G1 is not a primary co-activator of this nuclear receptor (data not shown). The possibility of NCU-G1 being a secondary co-activator was analysed by confocal fluorescence microscopy. HeLa cells were transiently transfected with expression vectors for EGFP-tagged NCU-G1 and RFP-tagged PPAR-alpha. As shown in figure 6B RFP-PPAR-alpha is predominantly localised in the nucleus as expected. In contrast, as shown in figure 6A the fusion protein NCU-G1-EGFP is mostly cytosolic. However, some NCU-G1-EGFP was found in the nucleus at distinct spots which might indicate transport into the nucleus. Once within the nucleus, as shown in figure 6C, the few NCU-G1-EGFP spots co-localise with RFP-PPAR-alpha showing potential cellular interaction domains for NCU-G1 and PPAR-alpha.

## Discussion

In this report, we describe the cloning and initial characterization of human NCU-G1. Sequence analysis detected no human homologues, however, NCU-G1 is highly conserved among species. Our experimental data show that NCU-G1 binds specifically to the FP1 element of the CRBP1 gene promoter and activates transcription from this promoter. Furthermore, NCU-G1 functions as a co-activator for PPAR-alpha directed reporter gene expression from the acyl-CoA oxidase promoter.

### NCU-G1 as a transcription factor

The identification of NCU-G1 as a DNA-binding protein suggested that it might be a transcription factor. In accordance with this notion we demonstrate specific binding to the FP1 element of the CRBP1 promoter. Further, NCU-G1 is shown to mediate activation of transcription from the CRBP1 promoter. This transcriptional activity requires the presence of the FP1 element, suggesting that this DNA element actually is a target site for this potential transcription factor. NCU-G1 is a small protein without recognizable transcription activation domains or DNA-binding domains, two important regions of a transcription factor. Deletion of various exons or segments of NCU-G1 indicated that exon 2 and exon 6 are important for transcriptional activity. Since this protein has no substantial homology to any characterized protein, novel regions with other activities may still exist, however, their identification and characterization will be the subject of a future project.

### NCU-G1 as a nuclear receptor coactivator

The promoter region of the human CRBP1 gene has a number of potential PPAREs. Furthermore, PPAR-beta agonists have been reported to stimulate CRBP1 expression [15]. The ability of NCU-G1 to stimulate PPAR transcriptional activity may therefore be relevant to CRBP1 expression. Another example of proteins with transcriptional potential via both DNA-binding and protein-protein interaction is the homeodomain transcription factor Prox1 [35]. These types of proteins, the activity of which can be modulated via multiple pathways, appear to play important roles in various transcriptional networks.

The detection of four LXXLL motifs in the primary sequence suggested that NCU-G1 might function as a nuclear receptor co-regulator. This hypothesis was explored in transient transfections, where NCU-G1 was found to strongly stimulate transcription from the acyl CoA-oxidase promoter, a well-known PPAR-alpha target [33]. This stimulation was shown to be ligand-dependent and therefore requires the conformational change of the AF2 domain occuring as a consequence of ligand-binding. Site-directed mutagenesis of the NCU-G1 NR-boxes clearly demonstrated that NR-box 1 plays a predominant role in PPAR-alpha stimulation, since its mutation completely eliminated NCU-G1's ability to co-activate PPAR-alpha. NR-box preference in the interaction between nuclear receptor and co-activator has been described for other co-activators [36,37]. NR-box 1 is present in all available sequences from man to frog, indicating that is important for NCU-G1 function.

Based on the analysis of flanking sequences from both naturally interacting partners and phage-display assays, NR-boxes have been organized into four classes according to the amino acids at position -1 and -2 (the N-terminal L in the LXXLL motif is arbitrarily designated as +1). All classes have a hydrophobic amino acid at position -1, except for motifs that belong to class I which have a basic amino acid at that position. A proline is usually present at position -2 in class II motifs whereas class III motifs have a serine or a threonine at that position. A basic amino acid is usually found at position -2 in class IV motifs [36-38]. According to these rules we find that NR-box 1 most likely belongs to class II, although it has a leucine in both position -1 and -2. In position -3 and -4 of this NR-box, a proline and a serine are present, respectively, making this a potential MAP-kinase target site, the phosphorylation of which may regulate interaction between NCU-G1 and nuclear receptors.

Mutation of any of the other three NR-boxes resulted in some reduction of reporter gene expression, hence it cannot be excluded that they too are of some importance to the NCU-G1 stimulated PPAR-alpha transcriptional activity. Alternatively, mutation of NR-boxes 2–4 may lead to conformational changes resulting in steric hindrance of the interaction between NR-box 1 and NCU-G1's protein partners.

Most co-regulators are large proteins with MW > 100 kDa. The 43.8 kDa NCU-G1 is much smaller, yet it is far from being the smallest of the presently known co-regulators, some of which are as small as 16 kDa [37]. Due to its small size and the fact that no domains with enzyme activity (such as histone deacetylase) or Q-rich domains mediating transcriptional activation have been identified in NCU-G1, we hypothesize that it may be a bridging factor. This notion fits with the presence of three potential SH3-binding domains. In other proteins such as MNAR, PXXP-motifs have been shown to mediate interaction with the SH3-domains in Src-kinases or STAT-proteins [14]. NCU-G1 may thus serve a function similar to that described for MNAR and PNRC which stabilize interaction between a Src-kinase and a nuclear receptor or transcription factor [28,39]. Such interaction between ER, c-Src and MNAR leads to phosphorylation of ER triggering stimulation of the transcriptional activation of ER target genes [13,14,37,38].

NCU-G1 has two NES but no recognizable NLS, yet endogenous NCU-G1 is clearly localized in the nucleus of the three cell types tested (figure 2). Lacking an identifiable NLS sequence, it is presently unclear how NCU-G1 translocates from cytosol to the nucleus. The NLS motif remains a poorly defined amino acid sequence [40]. Indeed, a number of proteins lacking known NLS have been shown to enter the cell nucleus [41]. In NCU-G1's case two mechanisms of nuclear translocation may be considered. One mechanism is based on size. Since NCU-G1 is a small protein with a molecular weight below the cut-off limit for free passage through nuclear pores, NCU-G1 may enter by simple diffusion. Once inside the nucleus, NCU-G1 may interact with a nuclear NES-masking protein serving to hold NCU-G1 in this compartment as has been reported for p53 [42]. The other mechanism is the so-called "piggy-back" mechanism where masking of the NES is involved in nuclear translocation as described for BRCA1. Here the interacting partner, BARD1 mediates transport into the nucleus [41]. Interaction between a nuclear receptor and the NES overlapping NR-boxes in NCU-G1 may also be regarded as a possible import mechanism for NCU-G1.

Based on NCU-G1's ability to co-activate PPAR-alpha we had anticipated direct interaction between the two proteins. Unfortunately, despite testing in several systems we failed to detect any direct interaction between the two proteins, indicating that NCU-G1 is not a primary co-activator for PPAR-alpha, but rather belongs to the group of secondary co-activators [34]. The results obtained by transient expression of the fluorescence tagged NCU-G1-EGFP and RFP-PPAR-alpha fusion proteins support this notion. In contrast to the localisation of endogenous NCU-G1 shown in figure 2, microscopy of ectopically expressed NCU-G1-EGFP showed extended localisation outside the nucleus. Fusion with EGFP leads to a molecular weight of approximately 70 kDa, well above the size limit for transfer to the nucleus by diffusion. Since NCU-G1 has no NLS this may explain why a large fraction of NCU-G1-EGFP is detected in the extranuclear compartment of the cell. The 3D-structure of NCU-G1 has not been solved, hence, we cannot exclude that fusion with EGFP disturbs the conformation of NCU-G1 regions important for subcellular trafficking. Although only a small part of expressed NCU-G1-EGFP enters the nucleus, co-localisation with RFP-PPAR-alpha is observed. Thus co-localised NCU-G1-EGFP is in a position to act as co-activator for PPAR-alpha.

## Conclusion

The present data suggest that NCU-G1 is a dual-function nuclear protein serving as a transcription factor as well as a nuclear receptor co-activator. Both these activities may contribute to controlling CRBP1 expression.

## Methods

### Plasmids

The pOTB7-hNCU-G1 from Origene (Rockville, MD, USA) was used as source of the ORF of NCU-G1. After digestion with EcoRI/Xho1 NCU-G1 was cloned into the Drosophila expression vector pAc5.1/V5-His vector (Invitrogen, CA, USA), creating pAc-NCU-G1. To create pNCU-G1-EGFP, the NCU-G1 ORF was excised by EcoR1/Age1 digestion from pOTB7-hNCU-G1 after modification of this plasmid by site-directed mutagenesis to insert an Age1 restriction site just before the stop codon of NCU-G1. The fragment was fused in-frame with EGFP in EcoR1/Age1 digested pEGFP-N1 (Clontech, CA, USA). The expression vector pRFP-PPAR-alpha was created by fusion in-frame of the Xho1/BamH1 digested mouse PPAR-alpha ORF, produced by PCR using the following primers: 5'-primer 5'-CTCCTCGAGCTATGGTGGACACAGAGA-3', 3'-primer 5'-TATGGATCCTCAGTACATGTCTCTGTAGA-3' and pSG5-mPPAR-alpha as template, into a Xho1/BamH1 digested pRFP-C1 vector (kindly donated by Dr. R. Tsien, Dept. of Pharmacology, University of California at San Diego, CA, USA [43].

#### NR-box mutations

Each NR-box LXXLL-sequence was mutated to LXXAA as described by the manufacturer using the QuikChange site-directed mutagenesis kit from Stratagene (La Jolla, CA, USA). The following primers were used (complementary antisense-strand sequences are not shown): NR-box 1 mutagenesis 5'-CCCCTGCTCCTTTGGACTGCAGCTCTGTTTGCAGCCCCA-3' creating pAc-NCU-G1#1mut, NR-box 2 mutagenesis 5'-GCCCCCTGCAGAACGCGGCTCATATACGGGCAGTGGGC-3' creating pAc-NCU-G1#2mut, NR-box 3 mutagenesis 5'-GTCTTCCAGTTGGACCAGGCAGCGTGGGGCTCCCTCCCA-3' creating pAc-NCU-G1#3mut, NR-box 4 mutagenesis 5'-GGCGGCTTGGTTCTGGCGGCGCACCACAAGAAGTACTCA-3' creating pAc-NCU-G1#4mut. The pAc-NCU-G1#1.4mut was constructed by mutation of all four NR-boxes.

#### Deletion mutations

All deletions were carried out as site-directed mutations according to the manufacturer using the QuikChange site-directed mutagenesis kit from Stratagene (La Jolla, CA, USA). Complementary antisense-strand sequences are not shown. Primer 5'-GAATTGCGGCCGTATGGTGGCCACCAAC-3' was used to create pAc-NCU-G1 (Δ aa1-81). Primer 5'-GACCCGCCAGGTGCTGCTTGAGTTTGAC-3' was used to create pAc-NCU-G1 (Δ exon 2). Primer 5'-CTTGTTTTTACCAGGTAAGGCCCGCTCTCTGG-3' was used to create pAc-NCU-G1 (Δ exon 3-6). Primer 5'-GGCCTTCAGGTAAGGCCCGCTCT-3' was used to create pAc-NCU-G1 (Δ exon 4-6). Primer 5'-GCCGTCTTCCATAAGGCCCGCTCTCTGG-3' was used to create pAc-NCU-G1 (Δ exon 5-6). Primer 5'-CTGGGTGTGGGCTAAGGCCCGCTCTCTGG-3' was used to create pAc-NCU-G1 (Δ exon 6). Primer 5'-CCCCACTAGTCCTGCACCACAAGAAG-3' was used to create pAc-NCU-G1(Δ aa372-394). All mutations were verified by DNA sequencing. The LacZ expression plasmid pAc5/V5.1-His/LacZ was obtained from Invitrogen. The pACO-CAT vector was a gift from Dr. Jonathan Tugwood [32] and pMT-mPPAR-alpha vector was a gift from Dr. Hilde Nebb, University of Oslo. The reporter plasmids expressing CAT under the control of various fragments of the hCRBP1 promoter cloned into the pOCAT1 vector have been described [17]. The reporter plasmid phCRBP1-CAT was constructed by insertion of a Kpn1/Xho1-digested PCR amplified CRBP1 promoter (-567/+104) fragment into the pCAT3 basic vector (Promega, Wi, USA). The CRBP1 promoter fragment was amplified using primers with the following sequences: forward primer 5'-CCGGTACCGAATTCCTGACCTCAGGTGATC-3', reverse primer 5'-GGCTCGAGTTCGGGGAGTGACTGGAGCCA-3'. The template was pA21EE, kindly donated by Magnus Nilsson [44].

### Cell cultures

JEG3 cells (36-HTB; ATCC, Boras, Sweden) were grown in MEM with Earle's salts (BioWhitaker, Verviers, Belgium) containing 1 g glucose/liter and supplemented with L-glutamine (2 mM), pyruvate (1 mM), non-essential amino acids, 10% foetal calf serum (FCS) and antibiotics (penicillin 100 units/ml, streptomycin 100 μg/ml). Flp-In 293 cells (Invitrogen, CA, USA) were grown in DMEM (high glucose) supplemented with L-glutamine (2 mM), 10% FCS and antibiotics (penicillin 50 units/ml, streptomycin 50 μg/ml, 100 μg Zeocin/ml). RPE cells (hTERT-RPE-1, Clontech, CA, USA) were grown in DMEM:F-12 supplemented with glutamine (2 mM), 10% FCS and antibiotics (penicillin 100 units/ml, streptomycin 100 μg/ml). HeLa cells were grown in the same medium as Flp-In 293 cells but without Zeocin. All mammalian cells were grown at 37°C in a humidified atmosphere under 5% CO_2_. Drosophila Schneider S2 cells (Drosophila Schneider line 2, # CRL-1963, ATCC) were grown in TC100 medium (PAA Laboratories, Coelbe, Germany) supplemented with 10% heat inactivated FCS (PAA laboratories, #A11-081, insect cell pretested), and antibiotics (penicillin 100 units/ml, streptomycin 100 μg/ml, and fungizone 0.25 μg/ml). The cells were grown at 26°C without CO_2_.

### Electrophoretic mobility shift assay

Double-stranded oligonucleotides representing the FP1 element in the CRBP1 promoter were labelled with [^32^P]γATP. For each reaction 3 × 10^4 ^cpm labelled probe was incubated with 2 μg GST-NCU-G1 in the presence or absence of competing oligonucleotides as described [17]. Oligonucleotide sequences were the following (only the upper strand is shown): FP1: 5'-GATCCGCCCGCTTGTGGCCAACTGGCTCCAGTCAC-3', AP1: 5'-CGCTTGATGACTCAGCCGGAA-3', Sp1: 5'-GATCATATCTGCGGGGCGGGGCAGACACAG-3', NF1: 5'-GATCTTATTTTGGATTGAAGCCAATATGAG-3'.

### Production of GST-NCU-G1 fusion protein

The ORF of NCU-G1 was excised from pOTB7-hNCU-G1 with EcoRI and Xho1 and fused in-frame with glutathione-S-transferase in the pGEX-5X-3 vector (GE Healthcare Bio-Sciences, Uppsala, Sweden). The fusion-protein was expressed in E. coli BL21(DE3) and purified according to the manufacturer's protocol (Invitrogen, CA, USA).

### Western analysis

Cells were homogenized and fractionated into nuclear and cytosolic fractions by the method of Dignam [45]. Twenty micrograms proteins were fractionated on a 10% SDS-polyacrylamide gel (29:1) and electro-transferred to a PVDF membrane (Bio-Rad, Hercules, USA). The membrane was blocked for 2 hrs in phosphate buffered saline containing 5% non-fat milk (PBSM). Primary antibody incubation for 2 hrs at 25°C was carried out with the NCU-G1 antiserum diluted 1:1000 in PBSM containing 0.1% Tween 20 (PBSMT). Membranes were washed in PBS containing 0.1% Tween 20 3 × 5 min., plus 3 × 15 min. with shaking. The secondary antibody (peroxidase-coupled goat-antirabbit IgG, GE Healthcare Biosciences, Uppsala, Sweden) was diluted 1:3000 in PBSMT and incubated with the membrane for 2 hrs at 25°C. Membranes were washed as described above and subjected to a final wash with PBS. Protein bands were visualized using Supersignal West Pico reagents from Pierce (Rochford, USA). The membranes were stripped by incubation for 5 min in 0.2 M NaOH followed by several rinses in PBS before further analysis.

### Northern hybridization

Hybridization was carried out on a human multi-tissue mRNA filter (Clontech, CA, USA) using a ^32^P-labeled NCU-G1 cDNA and beta-actin cDNA as a probes, according the manufacturer's instructions. NCU-G1 mRNA expression levels for each tissue were normalized to the expression levels of beta-actin and quantified by relative comparison of the intensity of ^32^P-labeled NCU-G1 cDNA.

### One-hybrid cloning

The MATCHMAKER One-hybrid System (Clontech, CA, USA) was used to clone NCU-G1. The following oligonucleotide sequence was used as bait: CGCTTGTTTTTTTCTGGCTC. This sequence is a modified version of the FP1 element from the hCRBP-1 gene promoter [17] from which the Sp1 binding site has been truncated and the NF1 binding site mutated to avoid interference from these transcription factors. A triplicate version of the oligonucleotide (CGCTTGTTTTTTTCTGGCTCCGCTTGTTTTTTTCTGGCTCCGCTTG-TTTTTTTCTGGCTC) was inserted into the EcoR1-Sac1 site upstream of the *HIS3 *reporter gene in the pHISi-vector. A MATCHMAKER cDNA library from human placenta (Clontech, CA, USA) was used for screening. Three positive clones were obtained one of which contained an ORF of 1221 bp. Sequencing was carried out by GATC (Konstanz, Germany) using the Sanger dideoxy method.

### Transfections

For transient transfections of S2 cells, 4 × 10^5 ^cells were plated in 9 cm^2 ^wells (6-well plates) in 2 ml complete medium. Transfection was carried out the following day using Fugene 6 (Roche, Mannheim, Germany). Each tissue culture well received a total of 1 μg DNA, comprising 250 ng pAc5.1-LacZ (Invitrogen, CA, USA), 350 ng reporter vector either pACO-CAT, phCRBP1-CAT or phCRBP1OCAT, 5–300 ng pMT-PPAR-alpha and 10 ng of pAc-NCU-G1 wild-type or mutated/truncated. When expressing PPAR-alpha, CuSO_4 _was added to 5 μM at the time of transfection to activate expression from the metallothionine promoter. Twelve hours after transfection, the PPAR-alpha agonist WY14643 (Sigma-Aldrich, St. Louis, USA) was added to 20 μM. Fifty-six hours after transfection the cells were harvested in 1× lysis buffer from the CAT ELISA kit (Roche, Mannheim, Germany). Expression of CAT was determined using this kit according to the manufacturer's instructions. The transfection efficiency was determined by measuring the γ-galactosidase activity. Reporter gene expression was calculated as pg CAT/unit β-galactosidase/hour and presented as relative expression levels compared to controls which were assigned the value 1.0. The data presented are the mean values of three separate transfections carried out in triplicates.

For confocal fluorescence microscopy HeLa cells were plated in microwell dishes for microscopy (Mattek, MA, USA) and transfected the following day using Lipofectamine according to the manufacturer's protocol (Invitrogen, CA, USA). Each tissue culture plate received 0.8 μg of pNCU-G1-EGFP and 0.8 μg of pRFP-PPAR-alpha. Microscopy was carried out 20 hours later.

### Antibodies

A peptide comprising the 15 C-terminal amino acids of hNCU-G1 was chosen for antibody production. Peptide sequence: LLLHHKKYSEYQSIN. Peptide synthesis, coupling of the peptide to KLH (keyhole limpet hemocyanin), injection into rabbits and bleeding was carried out by MedProbe/Eurogentec (Oslo, Norway). The antisera were tested in our laboratory. Preimmune serum did not detect any proteins in a Western analysis of crude nuclear extracts from transfected S2 cells transiently expressing NCU-G1. The antiserum detected a protein of approximately 45 kDa. The polyclonal antibodies directed against AP1, Sp1 and NF1 were purchased from Santa Cruz (Santa Cruz, CA, USA).

### Yeast two-hybrid interaction

Interaction between PPAR-alpha and NCU-G1 was assayed using Clontech's Matchmaker GAL4 Two-Hybrid System. Full-length mPPAR-alpha was PCR-amplifed using the following primers: forward primer 5'-TCGACCTTCCCGGGGATCCTGTCTCGAGAAATGGTGGACACGGAAAGCCCA-3', reverse primer 5'-ACTGAGTCGTCGACGCGGCCGCTTACTAGTACATGTCCCTGTAGATCTCCTG-3' and pMT-mPPAR-alpha as template. PPAR-alpha was digested with BamH1/Sal1 and fused in-frame with the GAL4 activation domain in BamH1/Xho1 digested pGADT7 creating pGADT7-PPAR-alpha. Full-length hNCU-G1 was PCR-amplified using the following primers: forward primer 5'-TCGACCTGAATTCATCCCGGGATCTCGAGTTATGCGCGGCTCTGTGGAG-3', reverse primer 5'-ACTGAGTCGTCGACGCGGCCGCTTACTAATTTATGGACTGGTACTCTGAGTA-3' and pOTB7-hNCU-G1 as template. NCU-G1 was fused in-frame with the GAL4 binding domain in pGBKT7 after digestion with EcoRI/Sal1 creating pGBKT7-NCU-G1. The authenticity of PPAR-alpha, NCU-G1 and their fusion sites were verified by sequencing. The yeast strains Y187-alpha transformed with pGADT7-PPAR-alpha and AH109a transformed with pGBKT7-NCU-G1 were mated by o/n co-incubation in synthetic complete medium (Clontech) lacking Leu and Trp before plating onto agar dishes of the same medium. Positive clones were transferred to agar dishes containing PPAR-alpha ligand (WY14643, 200 nM) but lacking Leu, Trp and His to test interaction between PPAR-alpha and NCU-G1. Y187-alpha transformed with pGADT7-T and AH109a transformed with pGBKT7-53 were used for positive controls. Y187-alpha transformed with PGADT7 and AH109a transformed with pGBKT7 were used for negative controls.

### Confocal fluorescence microscopy

Prior to imaging, the cell medium was changed to DMEM without phenol red and sodium carbonate (BioWhitaker, Verviers, Belgium). Image acquisition was performed on an Olympus Fluoview 1000 at 37°C with an Olympus PlanApo 60×/1.42 oil objective (Olympus Europa, Hamburg, Germany). More than 200 live cells from 5 transfections were analysed, selecting a wide variety of expression levels and the co-localisation conclusion is based on this.

## List of abbreviations

AF2 activation function domain 2

AP1 Activating protein 1

Bp1 binding protein 1

Bp2 binding protein 2

cAMP adenosine 3',5'-cyclic monophosphate

CAT chloramphenicol acetyl-transferase

CRBP1 cellular retinol-binding protein 1

CRM1 exportin chromosome region maintenance 1

EGFP enhanced green fluorescent protein

FP1 footprint 1

GST glutathione-S-transferase

NCU-G1 kidney predominant protein

NES nuclear export signal

NF1 nuclear factor 1

NLS nuclear localization signal

NR nuclear receptor

PPAR peroxisome proliferators-activated receptor

PVDF polyvinylidene difluoride

RAR retinoid acid receptor

RBP retinol-binding protein

RFP red fluorescent protein

RXR retinoid × receptor

SH3 Src-homology 3

Sp1 specificity protein 1

## Authors' contributions

KS cloned and sequenced hNCU-G1. He analysed the mRNA levels of NCU-G1 in human tissues, interaction between PPAR-alpha and NCU-G1 in a mammalian two-hybrid system and contributed to writing of the manuscript. MB carried out plasmid constructions and transient transfections of S2 cells. FS and OB analysed interaction between NCU-G1 and PPAR-alpha by confocal fluorescence microscopy. AK analysed subcellular localisation of NCU-G1 by Western. CK and VM analysed interaction between PPAR-alpha and NCU-G1 in yeast. SG initially identified Bp1 and Bp2 and contributed to writing of the manuscript. WE designed the project, wrote the manuscript, carried out *in silico *analysis of NCU-G1 and analysed NCU-G1 binding to DNA in EMSA. All authors have read and approved the final manuscript.
